# A Multi-Cell Hybrid Approach to Elevate the Energy Absorption of Micro-Lattice Materials

**DOI:** 10.3390/ma13184083

**Published:** 2020-09-14

**Authors:** Lijun Xiao, Xiao Xu, Weidong Song, Menglei Hu

**Affiliations:** 1 State Key Laboratory of Explosion Science and Technology, Beijing Institute of Technology, Beijing 100081, China; 7520180051@bit.edu.cn (L.X.); hmlbit2017@163.com (M.H.); 2 School of Mechatronical Engineering, Beijing Institute of Technology, Beijing 100081, China; xuxiao_1990@126.com

**Keywords:** micro-lattice structure, multi-cell hybrid, energy absorption, finite element modeling, additive manufacturing

## Abstract

Multi-cell hybrid micro-lattice materials, in which the stretching dominated octet cells were adopted as the strengthen phase while the bending dominated body centered cubic (BCC) lattice was chosen as the soft matrix, were proposed to achieve superior mechanical properties and energy absorption performance. Both stochastic and symmetric distribution of octet cells in the BCC lattice were considered. The cell assembly micromechanics finite element model (FEM) was built and validated by the experimental results. Accordingly, virtual tests were conducted to reveal the stress–strain relationship and deformation patterns of the hybrid lattice specimens. Meanwhile, the influence of reinforcement volume fraction and strut material on the energy absorption ability of the specimens was analyzed. It was concluded that the reinforced octet cells could be adopted to elevate the elastic modulus and collapse strength of the pure BCC micro-lattice material. The multi-cell design could lead to strain hardening in the plateau stress region which resulted in higher plateau stresses and energy absorption capacities. Besides, the symmetric distribution of reinforcements would cause significant stress fluctuations in the plateau region. The obtained results demonstrated that the multi-cell hybrid lattice architectures could be applied to tailor the mechanical behavior and plastic energy absorption performance of micro-lattice materials.

## 1. Introduction

Micro-lattice materials have attracted much attention due to their excellent mechanical properties, such as ultra-high specific stiffness and strength, outstanding vibration isolation performance and energy absorption capacities [1,2,3]. Different from the other lightweight cellular materials such as foams, whose microstructure is random and can hardly be designed to regulate the macroscopic properties, the micro-lattice materials are with periodic unit cells and the corresponding mechanical behavior can be easily tuned by the microscopic features. Especially with the rapid development of additive manufacturing, it becomes more convenient to precisely tailor the mesoscale parameters of micro-lattice materials for achieving better mechanical properties, such as higher modulus/strength and energy absorption [4,5,6]. Accordingly, numerous investigations have been conducted on different unit cells to explore the micro-lattice material with desired requirements.

The macroscopic mechanical performance of micro-lattice materials is closely related to their microscopic cell architectures. Depending on the nodal rigidity, Deshpande et al. [1] divided the lattice structures into two categories-bending dominated and stretching dominated. Ashby [2] systematically analyzed the effect of the two deformation mechanisms on the stress-strain response of cellular materials, concluding that the initial elastic modulus and collapse strength of stretching dominated lattices are much higher than those of the bending dominated lattices with the same relative density. However, the instability of struts in stretching dominated lattices will lead to local buckling and result in significant stress softening after the initial collapse strength when the relative density is low. Namely, the bending dominated lattices are suitable for energy absorption while the stretching dominate lattices are preferred for load bearing due to their high stiffness. Over the past decades, plenty of research on additively-manufactured micro-lattice materials with the above deformation mechanisms have been carried out. For instance, the bending dominated lattices with body centered cubic (BCC) cells [7,8], rhombic dodecahedron cells [9,10,11], diamond cells [12,13], and truncated cuboctahedron cells [12,14] were manufactured and their elastoplastic properties were experimentally tested. It was concluded that the bending dominated micro-lattice materials presented a long and flat plateau region which was applicable for energy absorption, but the stiffness and plateau stress were relatively low [7]. The most concerned stretching dominated cell is the octet truss structure [15,16,17,18], which is a face centered cubic (FCC) lattice and structurally more efficient than foams with a similar density made from the identical matrix material. It was reported that although the octet truss lattice with high relative densities possessed smooth plateau regions, significant stress fluctuations were observed in the lattice with low relative densities [18]. Accordingly, it is an important issue to improve both the elastic moduli and energy absorption capacity of the micro-lattice material with low density for lightweight design of protective structure.

Hybrid design is a common and effective approach to promote the properties of materials. For instance, the strength–ductility trade-off of metallic materials was overcome by transforming the single FCC crystal phase to FCC-HCP (hexagonal close-packed) dual-phase [19]. Meanwhile, the particle reinforced composite is also a kind of hybrid design by embedding the ‘hard’ particles into ‘soft’ matrix to elevate the strength and fatigue resistance of the material [20,21]. The hybrid concept can also be applied in the micro-lattice materials. Xiao et al. [22] proposed graded micro-lattice materials by the hybrid of cells with various sizes in different layers and achieved better impact resistance. The high elastic moduli and negative Poisson’s ratios were simultaneously obtained by Mirzaali et al. [23] through the random hybridization of different unit cell architectures. In their study, the auxetic and conventional hexagon unit cells were used as the basis cells. In another paper, the multi-material approach was adopted by Mirzaali et al. [24] to obtain the metamaterials with hard and soft phases. They concluded that the random assignment of a hard phase to original soft cellular materials could be applied to tailor the elastic modulus and Poisson’s ratio within a wide range. Maskery et al. [25] proposed a design approach for graded lattice structures by the hybridization of different surface-based cells, in which the structural weakening and high stress concentrations were solved. By using the multi-material printing process, Momeni et al. [26] investigated the mechanical performance of the hybrid octet truss lattices in which the exterior and internal elements were made of different materials. It was concluded that the exterior elements played a decisive role in the overall mechanical properties of the octet truss lattice, which could be employed to regulate the performance of the metamaterials. Multi-morphology sheet-based lattice structures were reported in [27], where the deformation mechanism was found to be shifted from layer-by-layer to the “shear band” mode and higher elastic properties were achieved compared to the solid-networks multi-morphology lattices. Kang et al. [28] proposed a multi-cell hybrid lattice structure based on topology optimization, and the newly designed structure was confirmed to exhibit higher stiffness as well as flexural rigidity compared to the solid material. Pham et al. [29] concluded that the hybrid of multiple unit cell architectures could be adopted to increase the damage-tolerance of micro-lattice materials. Most recently, multi-morphology lattices consisting of FCC and BCC unit cells were proposed by Alberdi et al. [30] and the plastic energy absorption performance was found to be improved compared with the lattice with uniform cells. The above investigations provide a new perspective on the optimal design of lattice structures by using the hybrid method. Nevertheless, either only the elastic properties of the hybrid lattices were focused on [23,24,27,28] or only the periodic assignment of sub-cells were considered [25,30].

Expanding upon the previous work, the present study aims to promote elastic moduli and energy absorption capacity of micro-lattice materials by the hybrid of multiple cell architectures. Inspired by the particle reinforced composites and dual-phase high entropy alloys with high strength and ductility, hybrid lattices combining ‘soft’ bending dominated cells and ‘hard’ stretching dominated cells are proposed to explore the feasibility of achieving the advantages of both. The ‘hard’ reinforcements are randomly distributed in the lattices and different volume fractions of reinforcements are considered. Virtual tests are carried out by finite element analysis to examine the mechanical response of the multi-morphology micro-lattices under compression and the detail deformation modes of the materials are presented. Afterwards, the effect of hybrid design on the elastoplastic behavior and energy absorption performance of the designed micro-lattices are revealed.

The paper is organized as follows. Section 2 introduces the choice of basic cells, the hybrid design strategy and the testing procedure used in this study. Section 3 presents the corresponding results obtained from the tests while some implications of the work are discussed in Section 4. Section 5 offers the concluding remarks and ideas for future work.

## 2. Materials and Methods

### 2.1. Material Design Principle

#### 2.1.1. Octet Truss Structure

**Figure 1:**
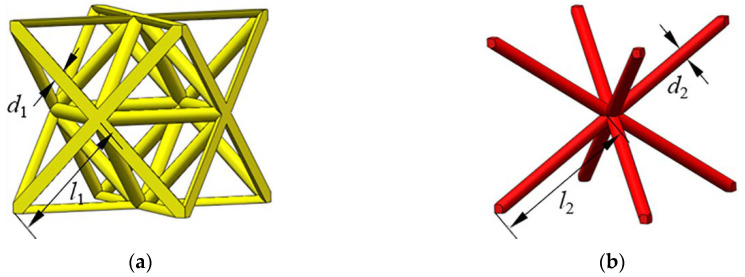
Schematic of the lattice structures: (**a**) octet lattice; (**b**) BCC lattice.

The octet truss structure as presented in Figure 1a is a typical orthotropic FCC structure derived from fundamental crystal lattices. According to Deshpande and Ashby [1], the octet lattice is a stretching-dominated structure which exhibits high specific stiffness and strength with a low relative density. However, the octet structure with low relative density is not stable and results in a significant decrease in stress after the first peak, which makes it inappropriate for energy absorption. The relative density ${\overset{-}{\rho }}_{oct}$ of an octet lattice structure can be expressed as

(1) $${\overset{-}{\rho }}_{oct}=\frac{3\sqrt{2}\pi }{2}{\left(\frac{d_{1}}{l_{1}}\right)}^{2}-6.825{\left(\frac{d_{1}}{l_{1}}\right)}^{3}$$

where *d*_1_ and *l*_1_ denote the diameter and the length of a single strut in the lattice structure. The second term represents the repeated volume at the joints and can be neglected when the aspect ratio of the strut is large. By performing the force analysis on a single strut using Timoshenko beam theory [16,31], the equivalent elastic modulus along the principal direction of the structure can derived to be

(2) $$\frac{E_{oct}}{E_{s}}=\frac{\sqrt{2}\pi d_{1}{}^{2}}{6l_{1}{}^{2}}\lambda_{1}\lambda_{2}$$

where *E_s_* is the elastic modulus of the matrix material. $\lambda_{1}=l_{1}/l_{1}{}^{\prime }$ represent the effect of the volume at the joints while $l_{1}{}^{\prime }$ is the effective length of a strut. $\lambda_{2}=1+\frac{3}{2}K_{T}{\left(\frac{d_{1}}{l_{1}{}^{\prime }}\right)}^{2}$ considers the bending deformation of the strut and *K_T_* is the shear modification of Timoshenko beam. When the relative density of the structure is small enough (i.e., the aspect ratio of the strut is large enough), the effects of the nodal volume and bending deformation can be ignored, then Equation (2) can be deteriorated to [15]

(3) $$\frac{E_{oct}}{E_{s}}=\frac{\sqrt{2}\pi d_{1}{}^{2}}{6l_{1}{}^{2}}=\frac{{\overset{-}{\rho }}_{oct}}{9}$$

The compressive strength of the structure can also be determined by

(4) $${\left(\sigma_{oct}\right)}_{s}=\{\begin{array}{cc} \frac{\sqrt{2}n^{2}\pi^{3}E_{s}}{32}{\left(\frac{d_{1}}{l_{1}}\right)}^{4}\lambda_{1}{}^{2}\lambda_{2} & \mathrm{when}\;\lambda_{1}\frac{d_{1}}{l_{1}}<\sqrt{\frac{16\sigma_{ys}}{n^{2}\pi^{2}E_{s}}}\;\mathrm{dominated}\;\mathrm{by}\;\mathrm{elastic}\;\mathrm{buckling}\\ \frac{\sqrt{2}\pi }{2}\sigma_{ys}{\left(\frac{d_{1}}{l_{1}}\right)}^{2}\lambda_{2} & \mathrm{when}\;\lambda_{1}\frac{d_{1}}{l_{1}}\geq \sqrt{\frac{16\sigma_{ys}}{n^{2}\pi^{2}E_{s}}}\;\mathrm{dominated}\;\mathrm{by}\;\mathrm{plastic}\;\mathrm{yielding} \end{array}$$

where $\sigma_{ys}$ is the yield strength of the basis material, *n* depends on the boundary condition of the strut which is 1 for pin-joint struts and 2 for fixed-end struts. Similarly, Equation (4) can be reduced to Equation (5) when the effects of the nodal volume and bending deformation are not considered

(5) $${\left(\sigma_{oct}\right)}_{s}=\{\begin{array}{cc} \frac{\sqrt{2}n^{2}\pi E_{s}}{144}{\overset{-}{\rho }}_{oct}{}^{2} & \mathrm{when}\;\frac{d_{1}}{l_{1}}<\sqrt{\frac{16\sigma_{ys}}{n^{2}\pi^{2}E_{s}}}\;\mathrm{dominated}\;\mathrm{by}\;\mathrm{elastic}\;\mathrm{buckling}\\ \frac{1}{3}{\overset{-}{\rho }}_{oct}\sigma_{ys} & \mathrm{when}\;\frac{d_{1}}{l_{1}}\geq \sqrt{\frac{16\sigma_{ys}}{n^{2}\pi^{2}E_{s}}}\;\mathrm{dominated}\;\mathrm{by}\;\mathrm{plastic}\;\mathrm{yielding} \end{array}$$

#### 2.1.2. BCC-Based Structure

BCC structure is a common crystal lattice in metal materials with high strength. The BCC micro-lattice structure consists of inclined struts dominated by bending deformation. Compared with the stretching-dominated octet lattice, the specific stiffness/strength of BCC lattice is much lower when both of them are with an incident high porosity. Nevertheless, the BCC structure is much more stable than the octet lattice which can lead to a long and flat plateau region for energy absorption. The relative density of BCC lattice structure can be determined by [8]

(6) $${\overset{-}{\rho }}_{BCC}=\frac{3\sqrt{3}\pi }{4}{\left(\frac{d_{2}}{l_{2}}\right)}^{2}-\frac{9\sqrt{2}}{4}{\left(\frac{d_{2}}{l_{2}}\right)}^{3}$$

where *d*_2_ and *l*_2_ denote the diameter and the length of a single strut in the lattice structure.

The equivalent elastic modulus of BCC lattice can be determined by [32]

(7) $$\frac{E_{BCC}}{E_{s}}=\frac{9\sqrt{3}\pi }{32}{\left(\frac{d_{2}}{l_{2}}\right)}^{4}$$

The compressive strength of BCC structure can be calculated by the implicit equation when considering the combination effect of bending moment and axial stress [33]

(8) $$\frac{F_{T}}{F_{T0}}=\frac{2}{\pi }\left[\arcsin\left(\sqrt{1-{\left(\frac{M}{M_{p}}\right)}^{2/3}}\right)+{\left(\frac{M}{M_{p}}\right)}^{1/3}\sqrt{1-{\left(\frac{M}{M_{p}}\right)}^{2/3}}\right]$$

where *F_T_* is the axial force loaded on the strut which can be expressed by the yield strength of the lattice material as $F_{T}=\sqrt{3}{\left(\sigma_{BCC}\right)}_{s}l_{2}^{2}/9$. *F_T_*_0_ represents the axial force related to the yield strength caused by pure normal stress, which can be calculated by $F_{T}{}_{0}=\pi d_{2}^{2}\sigma_{ys}/4$. *M* is the bending moment on the strut and can be expressed as $M=\sqrt{6}{\left(\sigma_{BCC}\right)}_{s}l_{2}^{3}/18$, while $M_{p}=\sigma_{ys}d_{2}^{3}/6$ is the plastic hinge. When the axial deformation is ignored, the expression can be reduced to

(9) $${\left(\sigma_{BCC}\right)}_{s}=\{\begin{array}{cc} \frac{3\sqrt{3}n^{2}\pi^{3}E_{s}}{64}{\left(\frac{d_{2}}{l_{2}}\right)}^{4} & \mathrm{when}\;\frac{d_{2}}{l_{2}}<\frac{32\sqrt{2}\sigma_{ys}}{3n^{2}\pi^{3}E_{s}}\;\mathrm{dominated}\;\mathrm{by}\;\mathrm{elastic}\;\mathrm{buckling}\\ \frac{\sqrt{6}\sigma_{ys}}{2}{\left(\frac{d_{2}}{l_{2}}\right)}^{3} & \mathrm{when}\;\frac{d_{2}}{l_{2}}\geq \frac{32\sqrt{2}\sigma_{ys}}{3n^{2}\pi^{3}E_{s}}\;\mathrm{dominated}\;\mathrm{by}\;\mathrm{plastic}\;\mathrm{yielding} \end{array}$$

As the struts in BCC lattice can be regarded as fixed-end beams, which leads to *n* = 2. Meanwhile, the yield strength of metallic material $\sigma_{ys}$ is usually much smaller than the Young’s modulus *E_s_*, which indicates that the struts in BCC lattice are dominated by plastic yielding.

#### 2.1.3. Hybrid Design

**Figure 2:**
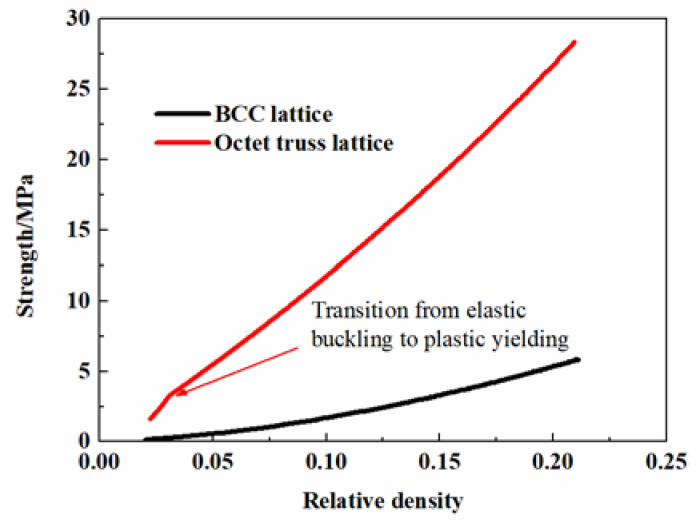
Comparison of strength between the octet truss lattice and BCC lattice structure.

**Figure 3:**
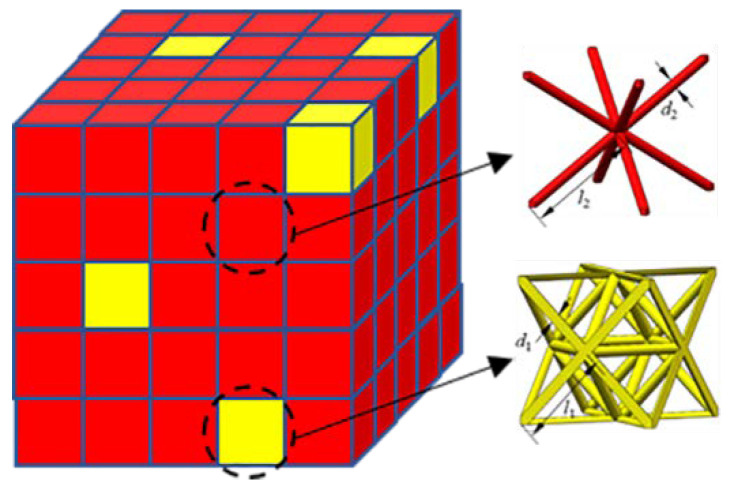
Schematic of the multi-cell hybrid lattice structures.

It can be concluded from the above analysis that the octet truss lattice is dominated by unstable buckling at low relative densities, which is not suitable for energy absorption. The BCC lattice structure is dominated by stable bending. Nevertheless, the collapse strength is related to the third power (or fourth power for elastic buckling) of the aspect ratio which is two orders higher than the octet truss lattice structure and the corresponding strength will be much lower. Figure 2 presents the comparison of initial strengths between the octet truss lattice and BCC lattice structure predicted by Equations (4) and (8), which indicates that the strength of the octet truss is almost one order of magnitude higher than the BCC lattice with the same low relative density. Accordingly, elevating the plateau strength of the BCC lattice or minimizing the unstable deformation of the octet truss lattice is an effective approach to promoting the energy absorption ability of the structure. In order to obtain the desired properties, the hybrid design method which usually combines the advantages of each constituent is considered here. The multi-cell hybrid lattice structure mixing the bending dominated and stretching dominated cell architectures are adopted as depicted in Figure 3. As the octet cell is much stronger than the BCC cell, which can act as the reinforced phase similar to the hard-soft dual-phase material design. Accordingly, the BCC lattice is adopted as the soft matrix and the octet cells are applied as the inclusions. Both the random distribution and symmetrical distribution of inclusions are taken into account. The random distribution of reinforcements is realized by MATLAB. A series of coordinates are generated stochastically and then the corresponding matrix cells are replaced by the inclusion cells. The volume fraction of the inclusion cells can be easily obtained by *V_f_* = *N_inc_*/*N_total_*, where *N_inc_* is the number of the inclusion cells and *N_total_* is the total cell number. The symmetrical hybrid structure can be directly built by ANSYS. Afterwards, the effects caused by the volume fraction of inclusions, as well as the distribution of inclusions, are examined by virtual tests.

### 2.2. Numerical Simulation Procedure

#### 2.2.1. Finite Element Model of the Micro-Lattice Structure

**Figure 4:**
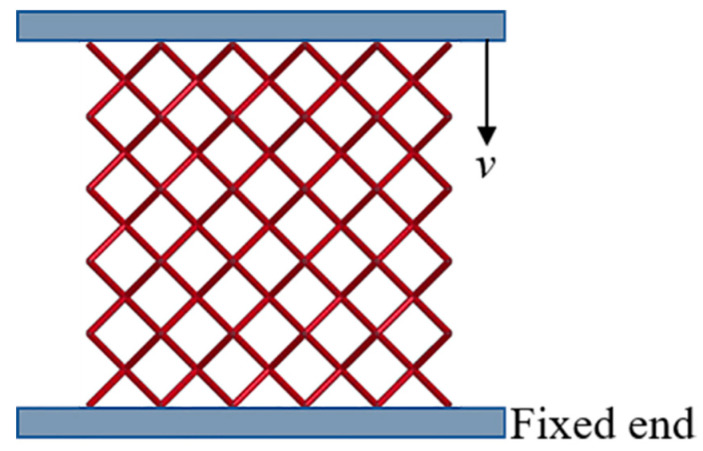
3D finite element model of the micro-lattice structure under compression.

A series of virtual tests were carried out by numerical simulation to examine the mechanical performance of the micro-lattice structures with different hybrid arrangements. The cell assembly mesoscopic finite element (FE) models were built by the commercial software Ansys/Lsdyna. Considering the low relative densities (i.e., large aspect ratio of the strut) of the micro-lattice structures in the present manuscript, the 3D beam element (i.e., beam161) with four integration points was used to mesh the micro-struts to reduce the computational cost. All the micro-lattice models contained five unit cells along each axis direction while the size of the unit cell was 8 mm × 8 mm × 8 mm. Each strut was meshed with five elements after inspecting the computational stability and mesh sensitivity. In all simulations, the micro-lattice structures were sandwiched between two rigid walls. The upper wall moved downward to compress the structure at a constant speed of 1 m/s while the other one kept stationary. The schematic of the finite element model is illustrated in Figure 4. The friction at the specimen–wall interface was ignored and a self-general-contact algorithm was adopted to consider the contact between the struts with large deformation. Four groups of inclusion volume fractions were considered in the simulations, i.e., *V_f_*(OCT) = 0, 0.04, 0.072 and 0.136 where *V_f_*(OCT) was the volume fraction of the octet cells in the hybrid lattice structures. The tests were repeated at least four times for each group of hybrid lattice model with randomly distributed octet reinforcements. Additionally, one more test was performed for *V_f_*(OCT) = 0.072 and 0.136 to account for the symmetric distribution of the reinforcements. All the simulations were performed on a high-performance desktop workstation with 32CPUs.

**Figure 5:**
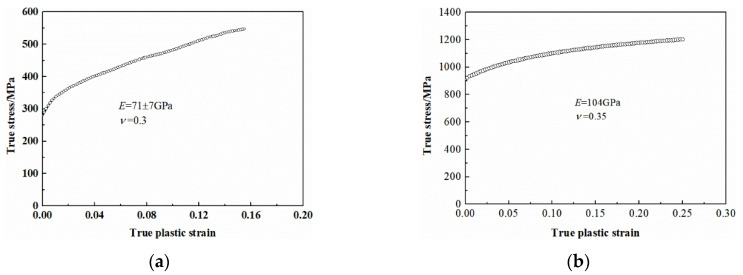
Stress–strain curves of the basis material: (**a**) for 316L stainless steel [34]; (**b**) for Ti-6Al-4V [35].

An isotropic hardening model which excludes the effect of anisotropic, temperature, and martensitic phase transformation was employed for simulation. In order to examine the effect of strut material on the simulation results, two metallic materials—i.e., 316L steel and titanium alloy Ti-6Al-4V—which had been widely applied in the field of energy absorber were assessed. Meanwhile, the additive manufacturing technology of these two materials had been quite mature which could avoid the negative influence caused by the fabrication instability. The parameters of additively manufactured 316L stainless steel and Ti-6Al-4V alloy obtained by previous experimental investigation [34,35] were adopted. The flow stress–strain curves of the materials are presented in Figure 5. The model “MAT_PIECEWISE_LINEAR_PLASTICITY” from the material library of Lsdyna, by which the flow stress–strain curve could be directly imported, was selected to simulate the material behavior. The density *ρ*, elastic moduli *E*, and Poisson’s ratio *ν* of 316L stainless steel are 7.93 × 10^3^ kg/m^3^, 71 ± 7 GPa, and 0.3 [34], while those of Ti-6Al-4V alloy are 4.43 × 10^3^ kg/m^3^, 104 GPa, and 0.35 [35], respectively. It should be noted that the adopted elastic modulus of 316L steel is much lower than that of the conventional material (~200 GPa). Li [34] attributed the discrepancy to the textured microstructure in the printed 316L steel caused by the selective laser melting process. Meanwhile, it has also been reported that the specimen size presents a significant influence on the mechanical properties of the additively-manufactured metallic materials [36,37]. As the tested specimen was with a small diameter of 220 μm similar to the strut thickness of the lattice structure, it might also lead to the lower elastic modulus than the conventional 316L steel. The failure of 316L steel was not considered as it was a kind of metallic material with high ductility. Meanwhile, our experimental results on the 316L steel-based BCC lattice specimens and the other investigations on the 316L steel based octet truss lattice structures [18] also revealed that the rapture was absent even though the specimens were compacted to densification. The SLM-printed Ti-6Al-4V alloy also exhibited a certain ductility although the strut fracture was observed according to our previous study [22]. Nevertheless, it should be noted that the beam element could not reflect the failure evolution properly. Considering that the structural design is the main concerned of our manuscript and in order to reduce the computational cost, the failure was not incorporated in the simulations for simplicity similar to [38].

#### 2.2.2. Validation of the Numerical Model

**Figure 6:**
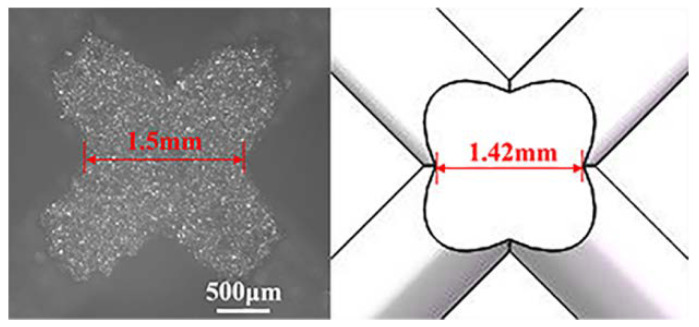
Comparison of geometry between the printed specimen and original model.

**Figure 7:**
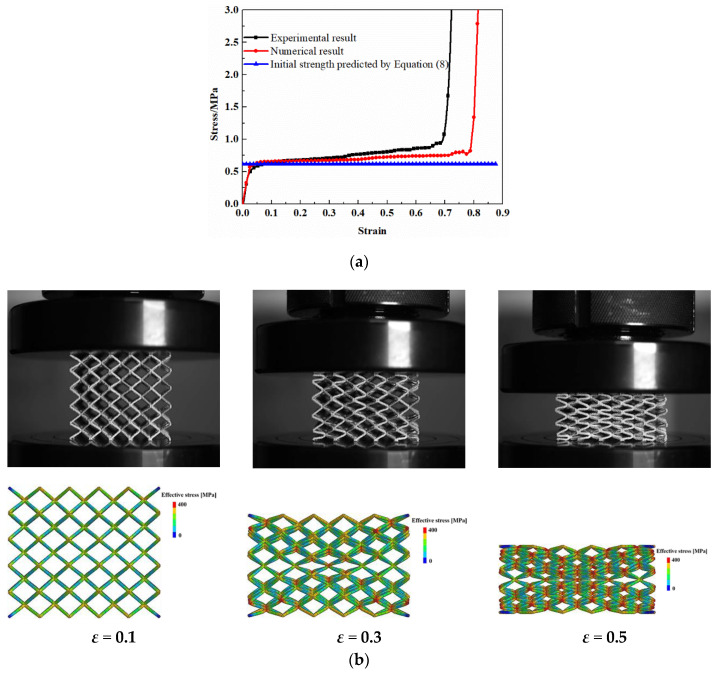
Validation of FE results: (**a**) stress-strain curves; (**b**) deformation mode.

The finite element modeling method was validated by the experimental data of SLM printed BCC micro-lattice material. The specimens with 5 × 5 × 5 BCC unit cells orthogonally stacked were manufactured on an SLM machine from Xi’an Bright Laser Technologies LTD, type BLT-S210, which was equipped with a disk laser with 260 W and the beam diameter was 60 μm. The adopted particles were with a diameter ranging from 15 μm to 53 μm. A standard scanning strategy was applied with a scanning speed of 1.2 m/s, while the layer thickness was 40 μm. As the particles were not pre-heated before printing, post-heat treatment was conducted on the fabricated lattice specimens to minimize the residual stress. The specimens were heated to 900 °C in a vacuum environment and kept for 2–4 h, which were subsequently cooled in the furnace filled with Argon gas. Sandblasting was conducted for about 5 min at an air pressure of 4 bars to remove the unmelted particles attached to the struts and reduce the roughness on the strut surface after manufacturing. Each BCC unit cell with a 8 mm edge length consisted of eight struts of 800 μm diameter, which results in an approximate relative density of 5%. The designed parameters and the real average parameters of the lattice specimens are listed in Table 1, which demonstrates the reliability of the printing process. The comparison of geometry at the joints between the printed specimen and original model is presented in Figure 6, which also supports that the discrepancy is insignificant. The micro-lattice specimens were compressed on an electronic testing machine (SUNS, UTM5504, Shenzhen SUNS Technology Stock Co., Ltd., Shenzhen, China) with a load cell of 100 kN under a constant velocity of 2.4 mm/min. The comparison between experimental and numerical results is presented in Figure 7. It can be observed that the numerical result exhibits a slightly larger densification strain than the experimental data, which is resulted from the limitation of beam element in dealing with the contact between the struts. The initial elastic modulus and plastic strength between the numerical and experimental results are quite close. The plateau stresses obtained from the numerical simulation and experiment are 0.7 MPa and 0.74 MPa, while the energy absorption capacities are 0.52 MJ/m^3^ and 0.50 MJ/m^3^, respectively (the detailed calculation is introduced in Section 4). Accordingly, it can be concluded that the discrepancy between the numerical and experimental results is in an acceptable range. Meanwhile, the deformation patterns captured by the numerical simulation and experiment are also in a good agreement, which validates the finite element model in the present work. Additionally, the analytical prediction on the initial strength by Equation (8) also matches well with the experimental and numerical results.

## 3. Results

**Figure 8:**
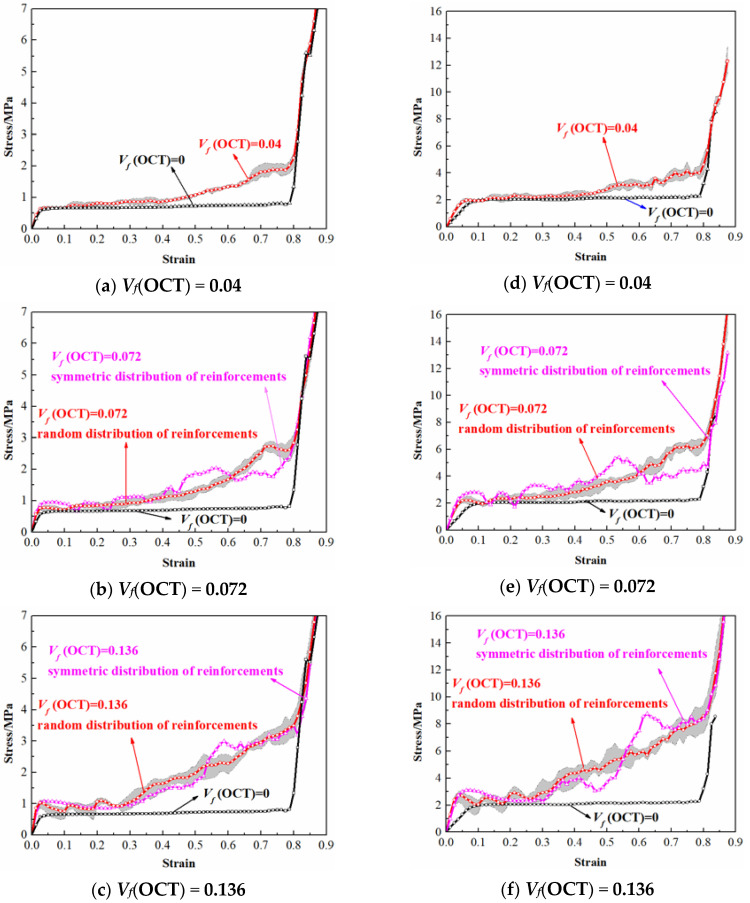
Numerical stress–strain curves of octet cell reinforced BCC lattices: (**a**–**c**) for the strut material of 316L stainless steel; (**d**–**f**) for the strut material of Ti-6Al-4V alloy.

In order to evaluate the effect of hybrid cell arrangement on the mechanical behavior of uniform lattice structures, a series of virtual tests on the lattices with different volume fraction of reinforcement cells were performed through finite element analysis. All the lattice specimens were maintained with an identical relative density of 0.05 by adjusting the strut diameter. The diameter of all the struts were uniform in the same lattice. The numerical results of the octet cells reinforced BCC lattices with different strut materials (i.e., 316L stainless steel and Ti-6Al-4V alloy) are depicted in Figure 8. The stress-strain curves are obtained by dividing the contact force between the upper rigid wall and lattice specimens with the area of the top diaphragm (40 × 40 mm^2^) and dividing the relative displacement with the initial height (40 mm) of micro-lattice material. Considering that excessive octet cell reinforcements may lead to unstable deformation as the stretching-dominated structures, the volume fraction of octet cells was controlled within 15%. As the spatial position of octet cells are randomly generated, each group is repeated at least for 4 times and the average value is regarded as the final result. The shadow regions in the images represent the error range of the numerical results.

**Figure 9:**
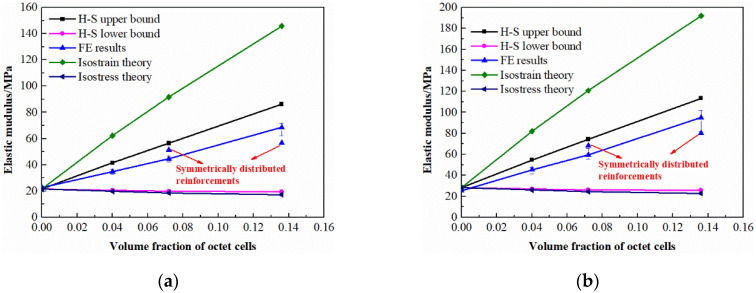
Variation of elastic modulus with different volume fractions of octet cells: (**a**) for the strut material of 316L stainless steel; (**b**) for the strut material of Ti-6Al-4V alloy.

**Figure 10:**
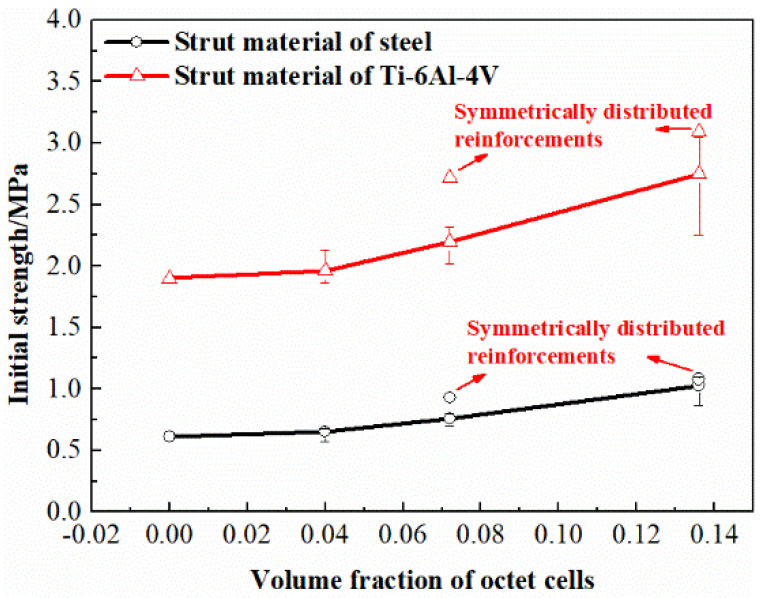
Variation of initial strength with different volume fractions of octet cells.

**Figure 11:**
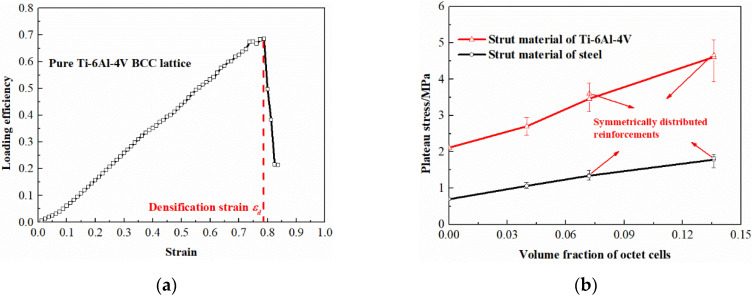
(**a**) Determination of densification strain; (**b**) plateau stress of different lattices.

It can be concluded from the results that the hybrid lattices with different strut materials exhibit similar stress-strain characteristics with the enhancement of octet cells. In common with the traditional cellular materials, the stress-strain curves of the multi-cell hybrid lattices can be divided into three parts. At the beginning of deformation, the stress and strain are linearly related which acts as the elastic behavior. Afterwards, a plateau region is observed where the cells starts to collapse and the stress remains unchanged or gradually increases with the increase of strain. Finally, the lattices are compacted together and the stress increases rapidly with the increase of strain, which is called the densification region. Apparently, the reinforcement of octet cells has a significant influence on the stress-strain response of BCC lattice structure. Firstly, the elastic modulus of the hybrid lattices are higher than the pure BCC lattice which are plotted in Figure 9. With the volume fraction of octet cells increases, the elastic modulus of the hybrid lattices increases almost linearly. Secondly, the octet cell reinforcements also affect the initial collapse strength of the lattices obviously. As exhibited in Figure 10, the initial collapse strengths of the hybrid lattices increase gradually with the enhancement of the octet cell number. Finally, the plateau regions of the hybrid lattices are also different from the lattice with uniform BCC cells. Unlike the flat plateau region of the pure BCC lattice, the hybrid lattices present a hardening behavior in plateau stress. Figure 11 presents the plateau stress of different hybrid lattices, which can be calculated by

(10) $$\sigma_{pl}=\frac{\int_{\varepsilon_{ys}}^{\varepsilon_{d}}\sigma \left(\varepsilon \right)d\varepsilon }{\varepsilon_{d}-\varepsilon_{ys}}$$

where *ε_ys_* is the strain corresponding to the initial collapse strength and *ε_d_* is the densification strain. *σ*(*ε*) is the stress-strain history during this strain interval. *ε_d_* is determined by the last peak of the loading efficiency curve *η*,

(11) $$\eta =\frac{\int_{0}^{\varepsilon }\sigma \left(\varepsilon \right)d\varepsilon }{\sigma \left(\varepsilon \right)}$$

which is also depicted in Figure 11.

Additionally, the differences between the lattices with randomly distributed reinforcements and symmetrical distributed reinforcements are also compared in Figure 9, Figure 10 and Figure 11. It can be observed that the elastic modulus of the lattices with symmetrical distributed reinforcements are higher than the lattices with stochastically distributed reinforcements when the volume fraction of octet cells is 0.072, while the situation is on the contrary when the volume fraction of octet cells is elevated to 0.136. For the initial collapse strength, the lattices with symmetrical distributed reinforcements are always slightly higher than those with stochastically distributed reinforcements. Nevertheless, the discrepancy in plateau stress of the two types of hybrid lattices is quite minor, which can be reflected from the stress-strain curves that the stochastic distribution of octet cells can lead to more smooth plateau regions as the stress fluctuations are more severe in the plateau regions of the lattices with symmetrical distributed reinforcements.

## 4. Discussion

### 4.1. Mechanism of Stress Elevation

The numerical results demonstrate that the reinforcement of octet cells can be adopted to elevate the elastic modulus of the pure BCC lattice. Usually, the elastic modulus of dual-phase composites can be predicted by several analytical models. The isostrain limit is an upper limit for composite modulus when each constitutive in the composite is subjected to the same strain, which can be expressed based on the rule of mixtures as [39]

(12) $$E=E_{oct}V_{f}(OCT)+E_{BCC}(1-V_{f}(OCT))$$

where *E* is the elastic moduli of the hybrid lattice. The isostress limit typically represents the lowest bound for composite modulus when each constitutive in the composite is subjected to the same stress, which can described by [39]

(13) $$\frac{1}{E}=\frac{V_{f}(OCT)}{E_{oct}}+\frac{1-V_{f}(OCT)}{E_{BCC}}$$

The Hashin–Strikman (H-S) bounds are the most widely used model to predict the elastic modulus of composites. As the hybrid lattice can be regarded as soft matrix stiffened by hard phase, the adjustable H-S upper and lower bounds can be adopted and determined by [40,41,42]

(14) $$E_{upper}=\frac{E_{oct}\left(E_{oct}V_{f}(OCT)+E_{BCC}(2-V_{f}(OCT))\right)}{E_{BCC}V_{f}(OCT)+E_{oct}(2-V_{f}(OCT))}$$

(15) $$E_{lower}=\frac{E_{BCC}\left(E_{BCC}(1-V_{f}(OCT))+E_{oct}(1+V_{f}(OCT))\right)}{E_{oct}(1-V_{f}(OCT))+E_{BCC}(1+V_{f}(OCT))}$$

where *E_upper_* and *E_lower_* represent the upper and lower bound of the elastic modulus, respectively. The comparisons between the FE results and theoretical predictions are presented in Figure 9. The elastic moduli of soft BCC matrix and hard octet reinforcement are approximately obtained from Equations (2) and (7). It can be concluded that the analytical elastic modulus of pure BCC lattice matches well with the numerical simulation results. Meanwhile, the elastic modulus of the multi-cell hybrid lattices fall in the H-S bound and are closer to the upper bound. It demonstrates that the multi-cell hybrid method can be adopted to improve the elastic modulus of micro-lattice materials.

**Figure 12:**
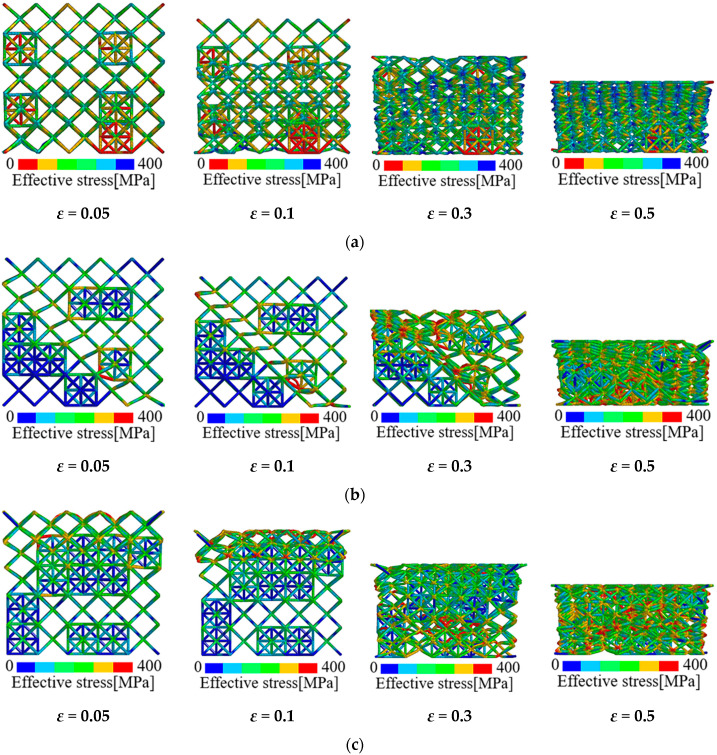
Deformation evolution of hybrid lattices with strut material of 316L stainless steel: (**a**) *V_f_*(OCT) = 0.04; (**b**) *V_f_*(OCT) = 0.072; (**c**) *V_f_*(OCT) = 0.136.

**Figure 13:**
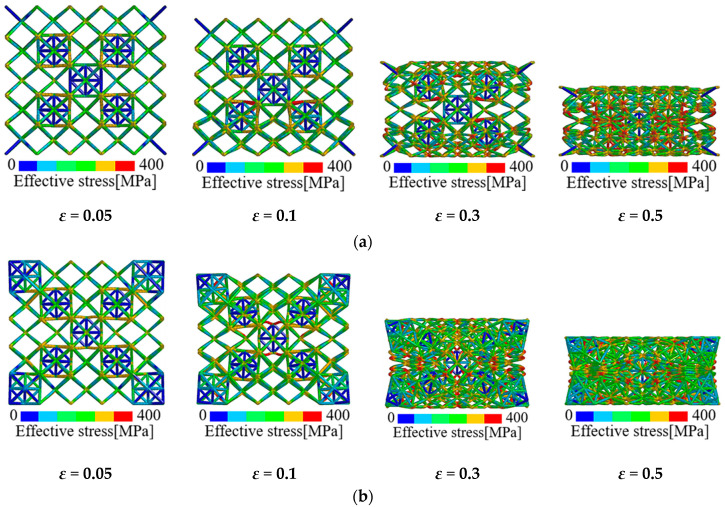
Deformation evolution of hybrid lattices with symmetrically distributed reinforcements: (**a**) *V_f_*(OCT) = 0.072; (**b**) *V_f_*(OCT) = 0.136.

Apart from the elastic modulus, the FE results also indicate that the initial collapse strength and plateau stress of the pure BCC lattice are elevated through hybrid design. Especially for the plateau stress, obvious strain hardening phenomenon is observed on the post-yielding stress-strain curves of hybrid lattices which is absent for pure BCC lattice. Actually in the multi-phase materials, such as particle reinforced composites [20,43] and dual-phase alloys [19], the hard inclusion phases provide the load-bearing capacity due to their high strengths while the soft matrix phases accommodate plastic deformation. Additionally, the existence of the hard phase will become the barrier to deformation which leads to the hardening on the post-yielding behavior of the material. This is similar to the effect of twinning on the plastic properties of metallic materials. In the present study, the octet cell phase has a higher density and strength than the BCC architected phase, which mainly contributes to the strength of the multi-cell hybrid lattices. Meanwhile, the plastic deformation of hybrid lattices is accommodated by the soft BCC cells. Figure 12 presents the deformation evolution of the multi-cell hybrid lattices with different volume fraction of octet cells. It can be observed that the deformation in the hybrid lattices is dominated by the soft BCC cells. With the loading proceeds, the octet cells start to deform and result in the elevation in stress. This phenomenon is consistent with the testing results on the layered dual-phase lattices by Pham et al. [29]. Compared with localization along the 45° plane in the pure BCC lattice exhibited in Figure 7, the reinforced octet cells in the hybrid lattices confine the movement of the joints which lead to the collapse of cells adjacent to the reinforcements. As the octet cells are stochastically distributed in the lattices, the deformation in the proposed hybrid lattices is also irregular. The deformation evolution of the multi-cell hybrid lattices with symmetrically distributed octet cells is exhibited in Figure 13. Apparently, the collapse of hybrid lattices with arranged reinforcements is also symmetric. Nevertheless, the periodic arrangements of cells may result in localized collapse and lead to the stress fluctuations in the corresponding plateau regions. It should be noted from Figure 13b that the symmetric hybrid lattice with *V_f_*(OCT) = 0.136 presents a concave deformation mode subjected to uniaxial compression, namely the negative Poisson’s ratio effect. This is resulted from the rotation of octet cells caused by the strength discrepancy between the soft and hard phase. Accordingly, the hybrid of bending dominated and stretching dominated cells can be adopted for tailoring the mechanical performance and deformation patterns of the micro-lattice materials.

### 4.2. Energy Absorption of the Hybrid Lattice Structures

As the micro-lattice material is widely applied as the energy absorption component in lightweight structural design, it is necessary to evaluate the energy absorption performance of the multi-cell hybrid lattices. The energy absorption capacity of the lattice material is calculated through the area surrounded by the stress-strain curve as

(16) $$EA=\int_{0}^{\varepsilon }\sigma \left(\varepsilon \right)d\varepsilon$$

**Figure 14:**
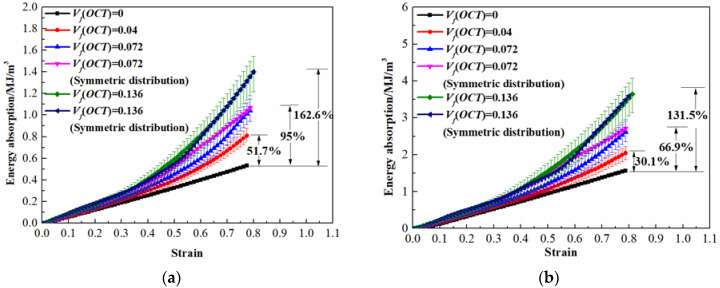
Energy absorption ability of hybrid lattices: (**a**) for the strut material of 316L stainless steel; (**b**) for the strut material of Ti-6Al-4V alloy.

When the integral upper limit is the densification strain *ε_d_*, the absorbed energy by the lattices reaches the maximum value. The energy absorption characteristics of the hybrid lattice structures are plotted in Figure 14. It can be obtained from Figure 14a that the absorbed energy by the octet cell reinforced BCC lattices increases with the enhancement of reinforcement amount. Compared with the pure BCC steel micro-lattice material, the maximum energy absorption increases by 95% when the fraction volume of octet cell is elevated to 0.072, and the enhancement can be 162.6% when *V_f_*(OCT) is 0.136. Similar promotion in energy absorption is also obtained for the Ti-6Al-4V hybrid lattices although the amplification is less significant. Regardless of the discrepancy in energy absorption during the loading process, it should be noted that the maximum energy absorption abilities of the hybrid lattices with symmetrical and stochastic distribution of reinforcements are quite close. The optimization of energy absorption performance is attributed to the hardening in plateau stress caused by the existence of octet cell reinforcements.

**Figure 15:**
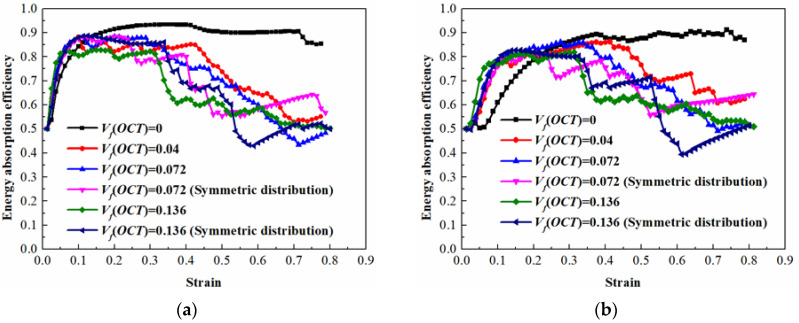
Energy absorption efficiency of hybrid lattices: (**a**) for the strut material of 316L stainless steel; (**b**) for the strut material of Ti-6Al-4V alloy.

In addition to the energy absorption capacity, the efficiency is also an important evaluation index. The energy absorption efficiency can be determined by

(17) $$EAF=\frac{\int_{0}^{\varepsilon }\sigma \left(\varepsilon \right)d\varepsilon }{\sigma_{\max}\varepsilon }$$

where *σ*_max_ is the maximum stress over the strain history. Figure 15 presents the energy absorption efficiency of the hybrid lattices with different strut materials and inclusion volume fractions. It can be concluded that the pure BCC lattices maintain the highest energy absorption efficiency in a long strain range due to their long and flat plateau stress. As the modulus of the hybrid lattices are higher than the pure BCC lattices, the corresponding energy absorption efficiency reaches the summit earlier and the values are almost at the same level of the pure BCC lattice. However, it should be noted that the duration of the maximum energy absorption efficiency turns out to be shorter due to the hardening phenomenon in the plateau stage.

## 5. Conclusions

The multi-cell hybrid micro-lattice architectures are proposed and their mechanical performance under uniaxial compression is investigated. Motivated by the dual-phase metallic materials and composites, the stretching dominated octet cells and bending dominated BCC cells are mixed to improve the energy absorption performance of the pure BCC micro-lattice material. The hybrid lattices are obtained by incorporating the hard octet cells into the soft BCC matrix stochastically and different volume fractions of octet cell inclusions are taken into consideration. Afterwards, virtual tests are conducted on the validated cell assembly finite element models to reveal the stress-strain relationship and deformation patterns of the hybrid lattice specimens. The influences of reinforcement volume fraction and strut material on the energy absorption ability of the hybrid micro-lattice materials are analyzed. Some conclusions drawn from the study are as follows:

(1) The multi-cell hybrid method can be adopted to tailor the mechanical behavior of micro-lattice materials. Compared with the pure BCC micro-lattice material, the addition of octet cells results in the higher elastic modulus, initial collapse strengths, and plateau stresses of the hybrid micro-lattice structures.
(2) With the volume fraction of octet cells increases, the elastic modulus of the hybrid lattices increase linearly. The elastic modulus of the multi-cell hybrid lattices are closer to the Hashin–Strikman upper bound.
(3) As the strength of the octet cells is much higher than the BCC cells, the initial collapse strength of the hybrid lattice increases gradually with the volume fraction of octet cells increases. Obvious strain hardening phenomena can be observed in the plateau stress stage of the hybrid lattices as the existence of the octet cell phase is the barrier to deformation.
(4) Resulting from the strain hardening in plateau stress, the energy absorption ability of the hybrid lattice is significantly improved compared with the pure BCC micro-lattice materials. The amplification of the maximum energy absorption can reach 162.6% for the steel lattice and 131.5% for the Ti-6Al-4V lattice when the volume fraction of octet cell is elevated to 13.6%.
(5) The effect caused by the distribution mode of octet cell reinforcements on the mechanical behavior of hybrid lattices is also detected. It is concluded that the symmetric distribution of octet cells will lead to significant stress fluctuations in the plateau stage, which is absent in the lattices with stochastic reinforcements. Nevertheless, the maximum energy absorption is hardly influenced by the distribution mode of the octet cells.
(6) Another interesting phenomenon is observed that when the octet cells are symmetrically distributed in the BCC lattice matrix with a volume fraction of 0.136, the hybrid lattice exhibits a ‘negative Poisson’s ratio’ deformation mode with high energy absorption capacity. This type of structure will be further investigated in the subsequent research due to its potential in defending technology.

## Figures and Tables

**Table 1:** Comparison between the designed and measured parameters

Parameters	Dimension (mm × mm × mm)	Mass (g)	Struct Thickness (μm)	Relative Density
Designed value	40 × 40 × 40	25.4	804	0.05
Measured value	40.36 × 40.32 × 40.52	27.8	830	0.053
